# Shirtsleeves to shirtsleeves in three research generations. A project on the science of science and the exceedingly rare circumstances that sustain research legacies from a mentor to mentee and to theirs: A case study on firearm research

**DOI:** 10.21203/rs.3.rs-4623060/v1

**Published:** 2024-07-23

**Authors:** Douglas J. Wiebe

**Affiliations:** University of Michigan

**Keywords:** public health, epidemiology, firearm injury prevention

## Abstract

**Background:**

In the 50 years since public health firearm research began, the decades have witnessed several pioneering investigators, followed by NRA backlash and a CDC funding moratorium, then increasing firearm mortality punctuated by mass shootings, and finally an unprecedented release of funding dedicated for research and to support trainees. Motivated by my own efforts to stay productive in firearm research, by the shirtsleeves-to-shirtsleeves cautionary lesson that wealth – for us this a researcher’s funding, infrastructure, and capacity – amassed by one generation will soon diminish, and by my worry that we are not adequately dedicated to growing new investigators, I set out to document researcher lineages in this field.

**Methods:**

I created a multigenerational lineage map to find authors using “gun” or “firearm” in the title/abstract as a way to find peer-reviewed publications on firearms as a public health issue. I designated the first author as Gen1 if the manuscript was sole authored or the senior author had never been first author on a firearm publication. I plotted each Gen1 author at the year of their first first-authored publication, and pointed from them to subsequent “first-time first-author investigators” (Gen2) for whom they were senior author, and so on for a Gen2 serving as senior author for a Gen3, and so on in that lineage.

**Results:**

Gen1 authors numbered 91 by 2023, the first being Rushforth in 1974.^3^ Rushforth, 14 years later, produced the first and his only Gen2 author, Paulson,^4^ who produced no Gen3 authors. The field had produced 6 Gen2 authors when the first Gen3 author appeared in 1993, who produced the first Gen4 author in 1998, 14 years after Kraus^5^ that initiated that lineage in 1984. To date, only 5 lineages have produced a Gen4 author and among those only one lineage, from Schwab in 2002,^6^ has produced a Gen5. Twenty-four Gen3 authors have emerged. Only 35% of Gen2 authors produced a Gen3.

**Conclusion:**

I hope this motivates years-long strategies to help trainees become established, informed by modeling quantitative and qualitative data to identify characteristics underlying the investigator network related to productivity and shortcomings alike. Without dedication to understand the science of science, shirtsleeves-to-shirtsleeves in three generations may be the fate of firearm research.

## BACKGROUND

2024 marks 50 years since public health firearm research began. The decades have witnessed several pioneering investigators publish the first groundbreaking studies, followed by NRA backlash that prompted a CDC funding moratorium,^1^ then increasing firearm mortality punctuated repeatedly by mass shootings, and finally an unprecedented release of foundation and federal funding dedicated for research to understand and prevent firearm-related harms and injury.

Indeed, 2013 had a watershed event when the NIH for the first time used “firearm” in a funding announcement, as the Obama administration answered public outcry after the Sandy Hook school shooting.^2^ The NIH, CDC, and NIJ have since allocated funding for R01, R03, and R21 research on firearms and notably T32 funding to train a new generation of firearm researchers.

Will more dedicated funding necessarily grow the field to have more firearm injury prevention researchers and more research? If we draw from economics and think of firearm grant dollars as wealth and investigators as businesses, we can think of investigators who hold grants as wealthy and thus having potential to produce output – research and trainees. Investigators hope their research will produce more publications. With more trainees, investigators can produce more publications, and with publications comes recognition, which helps win more grants.

Some investigators are sole proprietors and work independently, which limits how quickly they can produce, but not necessarily their longevity. Other investigators approach research like a business, engaging trainees who lead research the mentor oversees, yielding faster output for the mentor, and first outputs for mentees.

Hopefully investigators – with wealth to invest and a bent to pay it forward – have the simultaneous goal of helping trainees gain independence and secure funding of their own. Perhaps most investigators feel such a motivation; perhaps few know how to make it happen.

Legacy planning is fundamental to wealth management for individuals, who retain financial planners to help manage their investments, accumulate wealth, and live well while growing an inheritance to benefit their children and theirs. Enter “shirtsleeves-to-shirtsleeves in three generations,” the cautionary economics apothegm of how wealth accrued by one uniquely successful generation diminishes through two inheritance cycles, leaving eventual offspring no better off than their original ancestors.

Motivated by the potential for the field, lived experience in this fray of firearm research and funding, and my farmboy-turned-economist father who drew from the shirtsleeves-to-shirtsleeves lesson for planning family finances of his own, I set about documenting the timing, lineage, and fate of investigators and their mentees who entered this challenging field.

## METHODS

I created a multigenerational lineage map with PubMed to find authors using “gun” or “firearm” or related word in the title/abstract as a way to find peer-reviewed publications on firearms as a public health issue, and I designated the first author as Gen1 if the manuscript was sole authored or if the senior author had never been first author on a firearm publication. I plotted each Gen1 author at the year of their first first-authored publication, and then pointed from them to subsequent “first-time first-author investigators” (Gen2) for whom they were senior author, and so on for a Gen2 serving as senior author for a Gen3, and so on in that lineage. I thus identified pioneers, their mentees, their mentees’ mentees, and so on who entered the field.

## RESULTS

**Figure 1:**
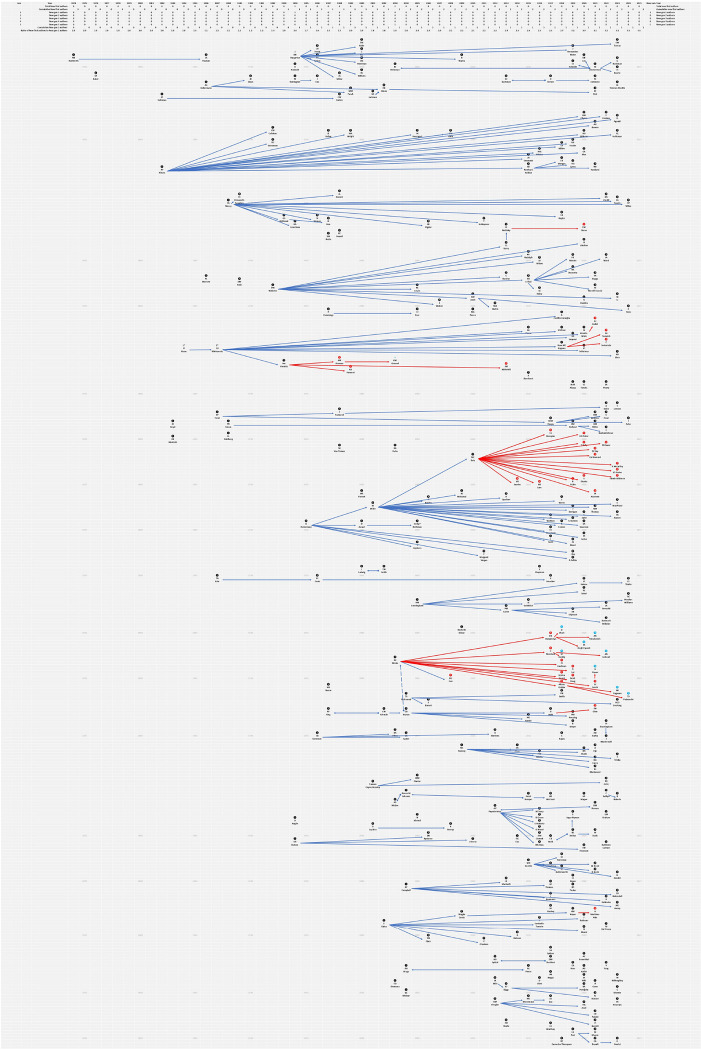
Firearm researcher lineage map with investigators listed at the year of their first first-authored firearm publication. Yearly timeline from 1974 through 2024 showing instances of an individual, in the year it occurred, for the first time being first-author on a peer-reviewed publication (Gen1 author) that reported on a public health or epidemiological topic or study of firearm injury and prevention. An arrow points from each of these individuals to any individual who, in the year it occurred, published their first first-authored firearm publication (Gen2 author) with the Gen1 author positioned as senior author. Additional arrows indicate Gen2 authors serving as senior author for Gen3 authors in the same lineage, and so on. Red arrows are used to indicate instances of a Gen3 author leading to a Gen4 (red number) author and to a Gen5 (blue number) author.

Gen1 authors numbered 91 by November 2023, the first being Rushforth in 1974 (Fig. 1).^3^ Gen1 authors increased from 2 by 1980 to 14 by 1990, 31 by 2000, 48 by 2010, and 79 by 2020. Rushforth, 14 years later, produced the first and his only Gen2 author, Paulson,^4^ who produced no Gen3 authors.

The field had produced 6 Gen2 authors when the first Gen3 author appeared in 1993, who produced the first Gen4 author in 1998, 14 years after Kraus^5^ that initiated that lineage in 1984. To date, only 5 lineages have produced a Gen4 author and among those only one lineage, started by Schwab in 2002,^6^ has produced a Gen5.

Rivara in 1992^7^ started what is the longest running lineage, producing 20 Gen2 authors at lags ranging from 10 to 40 years. The lineage with the highest Gen2/lag ratio was started by Papachristos in 2012,^8^ producing 6 Gen2 authors by 2016, no Gen3 authors to date.

Twenty-four Gen3 authors have emerged, most over the past 5 years. Only 35% of Gen2 authors produced a Gen3.

## DISCUSSION

Shirtsleeves to shirtsleeves in three generations has indeed been the norm for researcher lineages in our firearm injury prevention field. What should we take it to mean, seeing a Gen1 researcher produce no subsequent researchers, or seeing a researcher at any position in a lineage having no researcher offspring of their own?

Of course mentees are not offspring, and being senior author on someone’s first-authored manuscript does not necessarily indicate a mentor-mentee relationship. That measurement issue is one of several ways in which I limited my approach. Also, I did not capture the entire universe of relevant research, starting instead with prominent authors I new by reputation, and then snowball sampling among their co-authors to populate an incomplete yet quite comprehensive representation of the field. Also, my approach reveals my personal bias that we as investigators have the responsibility help trainees get started as firearm researchers, and to stay committed to helping them get firmly established and thrive as an investigator in this field. I feel that way in part because I have fabulous mentors and I want to pay it forward. Of course some trainees have mentors who are not helpful, and some mentors are outright harmful, and academic departments vary widely in what they require for faculty to be appointed and promoted. I have underway a much larger investigation into those issues and others, to determine the types of training and degrees trainees received (eg, MD, MPH, PhD), the types of departments and schools (eg, public health, sociology, criminology, medicine) and institutions (eg, R1 university, federal agency) where trainees trained and then got hired, the types of positions (eg, tenure track, research track) and expectations upon faculty as they started and pursed career stages (eg, R01 grants, high-impact publications), the numbers of and diversity among the mentors and peers and networks with whom they are connected, the availability of designated firearm research funding at times through one’s career, all to ultimately understand what has helped or has hampered efforts of the individuals who entered our field. Meanwhile, without delay, I am putting this forward to get a career planning conversation started for our field.

My hope is to motivate years-long strategies designed to help trainees become established, informed by modeling quantitative and qualitative data to identify characteristics underlying the investigator network related to productivity (eg, publications, grants, mentees, policy impact) and shortcomings (eg, career exit) alike. We may find that the most productive investigators have diverse portfolios, akin to sound financial planning; and that mentoring to achieve a legacy involves sustained supports for navigating wide-ranging circumstances at the right places and times. Fifty years in now, how far have we come, how efficient and strategic and responsible have we been, how far can we go? Without dedication to understand the science of science, shirtsleeves-to-shirtsleeves in three generations may be the fate of firearm research.

## Data Availability

Data will be shared upon reasonable request.

## References

[1] Rostron A, The Dickey Amendment on Federal Funding for Research on Gun Violence. A Legal Dissection. Am J Public Health. 2018;108(7):865–7. 10.2105/AJPH.2018.304450. 29874513 PMC5993413

[2] Bawagan J. US government targets gun research. CMAJ. 2013;185(9):E371–2. 10.1503/cmaj.109-4460. Epub 2013 Apr 15. 23589433 PMC3680571

[3] Rushforth NB, Hirsch CS, Ford AB, Adelson L. Accidental firearm fatalities in a metropolitan county (1958–1973). Am J Epidemiol. 1974;100(6):499–505. 10.1093/oxfordjournals.aje.a112062. 4447111

[4] Paulson JA, Rushforth NB. Violent death in children in a metropolitan county: changing patterns of homicide, 1958 to 1982. Pediatrics. 1986;78(6):1013–20. 3786026

[5] Kraus JF, Black MA, Hessol N, Ley P, Rokaw W, Sullivan C, Bowers S, Knowlton S, Marshall L. The incidence of acute brain injury and serious impairment in a defined population. Am J Epidemiol. 1984;119(2):186–201. 10.1093/oxfordjournals.aje.a113737. 6695898

[6] Sing RF, Branas CC, MacKenzie EJ, Schwab CW. Geographic variation in serious nonfatal firearm injuries in Pennsylvania. J Trauma. 1997;43(5):825 – 30. 10.1097/00005373-199711000-00015. 9390496

[7] Rivara FP, Stapleton FB. Handguns and children: a dangerous mix. J Dev Behav Pediatr. 1982;3(1):35–8. 7076862

[8] Papachristos AV, Braga AA, Hureau DM. Social networks and the risk of gunshot injury. J Urban Health. 2012;89(6):992–1003. 10.1007/s11524-012-9703-9. 22714704 PMC3531351

